# Association of TRAIL receptor with phosphatase SHP-1 enables repressing T cell receptor signaling and T cell activation through inactivating Lck

**DOI:** 10.1186/s12929-024-01023-8

**Published:** 2024-03-27

**Authors:** I-Tsu Chyuan, Hsiu-Jung Liao, Tse-Hua Tan, Huai-Chia Chuang, Yu-Chuan Chu, Meng-Hsun Pan, Chien-Sheng Wu, Ching-Liang Chu, Bor-Ching Sheu, Ping-Ning Hsu

**Affiliations:** 1 School of Medicine, National Tsing Hua University, Hsinchu, 30013 Taiwan; 2 Department of Medical Research, Cathay General Hospital, Taipei, 10630 Taiwan; 3 Department of Internal Medicine, Cathay General Hospital, Taipei, 10630 Taiwan; 4 Department of Medical Research, Far Eastern Memorial Hospital, New Taipei City, Taipei, 22000 Taiwan; 5 Institute of Biopharmaceutical Sciences, National Yang Ming Chiao Tung University, Taipei, 112304 Taiwan; 6 Immunology Research Center, National Health Research Institutes, Zhunan, 35053 Taiwan; 7 Department of Pathology and Immunology, Baylor College of Medicine, Houston, TX 77030 USA; 8 Department of Internal Medicine, Far Eastern Memorial Hospital, New Taipei City, Taipei, 22000 Taiwan; 9 Graduate Institute of Immunology, College of Medicine, National Taiwan University, Taipei, 10051 Taiwan; 10 Department of Obstetrics and Gynecology, College of Medicine, National Taiwan University Hospital, National Taiwan University, Taipei, 10002 Taiwan; 11 Graduate Institute of Clinical Medicine, College of Medicine, National Taiwan University, Taipei, 10051 Taiwan; 12 Department of Internal Medicine and Graduate Institute of Immunology, College of Medicine, National Taiwan University, 1 Jen-Ai Rd., Sec. 1, Taipei, 10051 Taiwan; 13 Department of Internal Medicine, National Taiwan University Hospital, Taipei, 10002 Taiwan

**Keywords:** TRAIL receptor, Phosphatase, SHP-1, T cell receptor signaling

## Abstract

**Background:**

T cell receptor (TCR) signaling and T cell activation are tightly regulated by gatekeepers to maintain immune tolerance and avoid autoimmunity. The TRAIL receptor (TRAIL-R) is a TNF-family death receptor that transduces apoptotic signals to induce cell death. Recent studies have indicated that TRAIL-R regulates T cell-mediated immune responses by directly inhibiting T cell activation without inducing apoptosis; however, the distinct signaling pathway that regulates T cell activation remains unclear. In this study, we screened for intracellular TRAIL-R-binding proteins within T cells to explore the novel signaling pathway transduced by TRAIL-R that directly inhibits T cell activation.

**Methods:**

Whole-transcriptome RNA sequencing was used to identify gene expression signatures associated with TRAIL-R signaling during T cell activation. High-throughput screening with mass spectrometry was used to identify the novel TRAIL-R binding proteins within T cells. Co-immunoprecipitation, lipid raft isolation, and confocal microscopic analyses were conducted to verify the association between TRAIL-R and the identified binding proteins within T cells.

**Results:**

TRAIL engagement downregulated gene signatures in TCR signaling pathways and profoundly suppressed phosphorylation of TCR proximal tyrosine kinases without inducing cell death. The tyrosine phosphatase SHP-1 was identified as the major TRAIL-R binding protein within T cells, using high throughput mass spectrometry-based proteomics analysis. Furthermore, Lck was co-immunoprecipitated with the TRAIL-R/SHP-1 complex in the activated T cells. TRAIL engagement profoundly inhibited phosphorylation of Lck (Y394) and suppressed the recruitment of Lck into lipid rafts in the activated T cells, leading to the interruption of proximal TCR signaling and subsequent T cell activation.

**Conclusions:**

TRAIL-R associates with phosphatase SHP-1 and transduces a unique and distinct immune gatekeeper signal to repress TCR signaling and T cell activation via inactivating Lck. Thus, our results define TRAIL-R as a new class of immune checkpoint receptors for restraining T cell activation, and TRAIL-R/SHP-1 axis can serve as a potential therapeutic target for immune-mediated diseases.

**Supplementary Information:**

The online version contains supplementary material available at 10.1186/s12929-024-01023-8.

## Background

Autoimmunity is characterized by the breakdown of immune tolerance resulting in tissue inflammation. T cells play a central role in regulating and developing autoimmunity. T cell homeostasis is highly dynamic and tightly controlled by internal signals from T cell receptors (TCRs). Immune checkpoint receptors are major gatekeepers that restrain TCR signaling to avoid autoimmune inflammation.

Tumor necrosis factor (TNF)-related apoptosis-inducing ligand (TRAIL) belongs to the TNF superfamily and has highly similar homology with the Fas ligand [1, 2]. TRAIL induces apoptotic signaling after binding to TRAIL receptor (TRAIL-R) [3, 4] to activate caspase-8 and the downstream caspase cascades [5]. Although the actual biological role of TRAIL remains unclear, accumulating evidence in recent years has demonstrated its role in modulating immune responses and T cell homeostasis in autoimmune diseases [6–10]. TRAIL profoundly inhibits T cell activation and suppresses the development of autoimmune diseases without inducing apoptosis in animal models [11–13]. These results indicate that the TRAIL/TRAIL-R interaction transduces signals that directly suppress T cell activation. However, the distinct pathway through which TRAIL-R transduces signals to directly inhibit T cell activation remains unclear.

In the present study, we explored a unique signaling pathway activated by TRAIL-R that directly inhibits T cell activation. We screened for intracellular TRAIL-R-binding proteins in T cells using immunoprecipitation coupled with mass spectrometry–based proteomics. We identified an association between TRAIL-R and the tyrosine phosphatase SHP-1, which transduces a co-inhibitory signal to inhibit proximal TCR signaling and subsequent T cell activation**—**a signaling pathway distinct from the conventional apoptosis signaling pathway**—**via inactivation of Lck. These findings suggest that TRAIL-R serves as a novel immune checkpoint receptor for the regulation of autoimmunity.

## Material and methods

### Experimental animals

TRAIL-R knockout mice (*Trail-r*^−/−^, C57BL/6 background) were provided by Prof. Henning Walczak (UCL Cancer Institute, University College London, United Kingdom) [14]. Wild-type C57BL/6 *Trail-r*^+/+^ (female, 6–7 weeks old) and *Trail-r*^−/−^ (female, 6–7 weeks old) mice were maintained and used in accordance with the guidelines of the Association for Assessment and Accreditation of Laboratory Animal Care at the National Taiwan University Medical Center. All experimental protocols were approved by the Animal Ethics Committee of the National Taiwan University Medical Center (IACUC Approval No: 20190025).

### Cells

CD4^+^ T cells were isolated from murine spleens using an EasySep Mouse CD4^+^ T cell Isolation Kit (STEMCELL Technologies, catalog no. 19852; Vancouver, BC, Canada). Murine CD4^+^ T cells and Jurkat T cells (American Type Cell Culture [ATCC]: TIB-152) were cultured in RPMI-1640 medium (Invitrogen, Carlsbad, CA), containing 10% fetal calf serum (Invitrogen), penicillin (10 units/mL; Invitrogen), and streptomycin (10 µg/mL; Invitrogen). EL4 T cells (ATCC: TIB-39) were cultured in DMEM medium (Gibco, Grand Island, NY) containing 10% horse serum (Gibco), penicillin (10 units/mL; Invitrogen), and streptomycin (10 µg/mL; Invitrogen).

### Plasmids, siRNAs, and transfection

To generate expression plasmids for flag-tagged full-length (1–381; TRAIL-R^WT^-FLAG) or death domain-truncated TRAIL-R mutant (1–273; TRAIL-R^△DD^-FLAG), the coding sequence for full-length and death domain-truncated derivatives of mouse TRAIL-R were isolated using RT-PCR, and the amplified product was ligated in-frame into pcDNA3.1 vector. The resulting plasmids were confirmed using DNA sequencing. The primers used for TRAIL-R^WT^-FLAG were 5′-CGTCAGATCCGCTAGCATGGACTACAAGGACGACGATGACAAGGAGCCTCCAGGACCCAGCA-3′ and 5′-GTCGACTGCAGAATTCTCAAACGCACTGAGATCCTC-3′; the primers for TRAIL-R^△DD^-FLAG were 5′-CGTCAGATCCGCTAGCATGGACTACAAGGACGACGATGACAAGGAGCCTCCAGGACCCAGCA-3′ and 5′-GTCGACTGCAGAATTCTCACTTCAGGTCGTCAGCTGAGT-3′. TRAIL-R^WT^-FLAG or TRAIL-R^ΔDD^-FLAG plasmids were transfected into EL4 T cells using electroporation. Briefly, EL4 cells (5 × 10^5^ cells) were gently mixed with the plasmid (10 µg) in an electroporation buffer (Neon Transfection System Kit, catalog no. MPK10096; Invitrogen), transferred to an electroporation cuvette, and electroporated using the Neon Transfection System (Invitrogen), following the manufacturer’s protocols. SHP-1 siRNA (catalog no. sc-29479), and control siRNA (catalog no. sc-37007) were purchased from Santa Cruz Biotechnology (Dallas, TX). For each transfection, 1 μg of siRNA duplex mixed with 5 μL siRNA transfection reagent (catalog no. sc-29528; Santa Cruz Biotechnology) was incubated with murine CD4^+^ T cells (2 × 10^5^ cells), according to the manufacturer’s protocol.

### Antibodies and reagents

Recombinant mouse TRAIL protein (catalog no. Z03367) was purchased from GenScript (Piscataway, NJ). Stimulatory anti-CD3 (catalog no. 100253) and anti-CD28 (catalog no. 102121) antibodies were purchased from BioLegend (San Diego, CA). The antibodies used for flow cytometry, including anti-CD69-FITC (catalog no. 104506) and anti-IL-2-PE (catalog no. 503808), were also purchased from BioLegend. Anti-CD25-PE (catalog no. 553866) antibody was purchased from BD Biosciences (Franklin Lakes, NJ). For immunoblotting and immunoprecipitation, anti-LCK (catalog no. 2752S), anti-ZAP70 (D1C10E, catalog no. 3165S), anti-p-ZAP70 (Y319/Y352, catalog no. 2717S), anti-PLCγ1 (D9H10, catalog no. 5690), anti-p-PLCγ1 (Y783, catalog no. 2821S), anti-p-LAT (Y255, catalog no. 45170), anti-PKCθ (E117Y, catalog no. 13643), anti-p-PKCθ (T538, catalog no. 9377), anti-p-IKKβ (S176/180, catalog no. 2697S), anti-JNK (catalog no. 9252), anti-p-JNK (T183/Y185, catalog no. 9251), anti-p38 mAPK (catalog no. 9212), anti-caspase 8 (11G10, catalog no. 9748S), anti-p-SHP-1 (Y564, catalog no. 8849S), anti-SHP-2 (D50F2, catalog no. 3397S), anti-p-SHP-2 (Y580, catalog no. 3703S), anti-CBL (catalog no. 2747S), anti-CSK (C74C1, catalog no. 4980), and anti-FLAG (DYKDDDDK Tag Antibody, catalog no. 2368) antibodies were purchased from Cell Signaling Technology (Danvers, MA). Anti-p-LCK (Y394, catalog no. 933102), anti-ERK1/2 (catalog no. 686901), and anti-p-ERK1/2 (T202/Y204, catalog no. 675506) antibodies were purchased from BioLegend. Anti-p-CD3ζ (Y142, catalog no. abx012434) antibody was purchased from Abbexa (Cambridge, UK). Anti-LAT (catalog no. PA5-82419) antibody was purchased from Invitrogen. Anti-TRAIL-R (catalog no. 3062) was purchased from BioVision (Milpitas, CA). Anti-CD3ζ (G3, catalog no. ab11281), Anti-IKKβ (EPR6043, catalog no. ab124957), anti-Caspase 3 (EPR18297, catalog no. ab184787), and anti-SHP-1 (catalog no. ab227503) antibodies were purchased from Abcam (Cambridge, UK). Anti-p38 MAPK (catalog no. 14064–1) and anti-p-p38 MAPK (T180/Y182, catalog no. 28796–1) antibodies were purchased from Proteintech (Rosemont, IL). Anti-flotillin-1 antibody (catalog no. 610821) was purchased from BD Biosciences (Franklin Lakes, NJ). Anti-β-actin (clone C4, catalog no. MAB1501) antibody was purchased from Merck Millipore (Billerica, MA). For confocal imaging, Lck-Cy3 was purchased from Bioss (catalog no. BS-2649R; Woburn, MA). Anti-ZAP-70-FITC (catalog no. 313404) was purchased from BioLegend. Anti-ganglioside GM-1 (catalog no. ab23943) was purchased from Abcam. All primary antibodies used for immunoblotting were used at 1:1000 dilutions. The immunoprecipitation antibodies were used at a dilution of 1:200. The pan-caspase inhibitor Z-VAD-FMK (catalog no. ab120487) was purchased from Abcam. All the antibodies used for the immunoblotting and immunoprecipitation were listed in Supplementary Table S1.

### Immunoblotting analysis

Total cell lysates were prepared on ice using PhosphoSafe Extraction Reagent (catalog no. 71296; Merck Millipore). The protein extracts were fractionated on SDS–polyacrylamide gel electrophoresis (SDS-PAGE) and transferred onto polyvinylidene difluoride membranes. The membranes were probed with individual primary antibodies, followed by incubation with horseradish peroxidase-conjugated secondary antibodies. The horseradish peroxidase substrate reaction was performed using a chemiluminescent reagent (catalog no. WBKLS0500; Merck Millipore), and the chemiluminescent signal was detected using iBright Imaging Systems (Invitrogen).

### Immunoprecipitation

Cell extracts were incubated with individual antibodies and protein G-agarose beads (catalog no. M16-266; Merck Millipore) in lysis buffer (50 mM Tris [pH 8.0], 150 mM NaCl, 1% Triton X-100, 0.5% deoxycholate, 0.1% SDS, 2 µg/mL leupeptin, 5 µg/mL aprotinin, 1 mM phenylmethylsulphonyl fluoride, 1 mM dithiothreitol, and 1 mM Na_3_VO_4_) with continuous rotation at 4 °C for 3 h. Immunoprecipitates were washed three times with lysis buffer before use in the immunoblotting analyses.

### Lipid raft isolation

Cell extracts were incubated with 0.2% Triton-X, 50 mM HEPES, 100 mM NaCl, 5 mM EDTA, 1% Ser/Thr protein kinase inhibitor, 1% Thr protein kinase inhibitor, and 1% protease inhibitor, for 1 h on ice. The lysate was diluted (1:1) with 80% ice-cold sucrose in 150 mM NaCl, 5 mM EDTA, and 25 mM MES; transferred to Ultra-Clear centrifuge tubes (Beckman Coulter, Brea, CA); and overlaid with ice-cold 30% sucrose followed by ice-cold 5% sucrose. The sucrose gradients were centrifuged at 200 000 × g in a Beckman Coulter SW41Ti rotor for 22 h at 4 °C. Twelve 375 μL fractions were collected and SDS-PAGE sample buffer was added to the harvested fractions before immunoblotting analyses.

### Flow cytometry

For the cell proliferation assay, cells were stained with carboxyfluorescein diacetate succinimidyl diester (CellTrace CFSE kit, catalog no. C34570; Thermo Fisher Scientific, Waltham, MA) with indicated treatment for 96 h followed by flow cytometry. For the intracellular staining of IL-2, the cells were pre-incubated with monensin (GolgiStop™, catalog no. BDB554724; BD Biosciences) for 4 h, followed by fixation and permeabilization with BD Cytofix/Cytoperm (catalog no. 554714; BD Biosciences) and staining with the indicated antibodies for 30 min on ice before flow cytometric analysis. For the apoptosis assay, cells were stained with a PE-Annexin V/7-AAD apoptosis detection kit (catalog no. 640934; BioLegend). The ratio of Annexin V^+^ cells to total cells indicated the percentage of apoptotic cells. All data were acquired using a FACSCanto II (BD Biosciences) and analyzed with FlowJo ver.10.8.1 software (BD Life Sciences, Ashland, OR).

### Enzyme-linked immunosorbent assays

The levels of IL-2 (ELISA MAX™ Deluxe Set Mouse IL-2, catalog no. 431004; BioLegend), IL-4 (ELISA MAX™ Deluxe Set Mouse IL-4, catalog no. 431104; BioLegend), and IFN-γ (ELISA MAX™ Deluxe Set Mouse IFN-γ, catalog no. 430804; BioLegend), from the cell lysates were analyzed by enzyme-linked immunosorbent assays (ELISAs) as described by the manufacturer (BioLegend).

### Confocal microscopy

Cells (2.0 × 10^6^) were incubated with anti-CD3 (3 µg/mL) and anti-CD28 (2 µg/mL) antibodies in the presence or absence of TRAIL (10 µg/mL) for 30 min at 37 °C. After washing with phosphate-buffered saline (PBS) at 4 °C, the cells were fixed with 4% formaldehyde for 1 h, permeabilized with 0.5% Triton X-100 in PBS for 15 min, and stained with the indicated antibodies. After washing with 1% bovine serum albumin in PBS, the cells were mounted on glass slides using a drop of Corning Matrigel matrix (catalog no. 354234; Corning Life Sciences, AZ). Images were obtained using a Leica SP8 STED confocal microscope (Wetzlar, Germany) with a 100 × objective lens. The laser sources used were a 405 nm diode laser and a 470–670 nm white-light laser.

### Liquid chromatography–tandem mass spectrometry (LC–MS/MS) and data analysis

Mass spectrometry was performed as described previously [15, 16]. Briefly, protein samples were separated using SDS-PAGE and stained with Coomassie Brilliant Blue. The protein bands were then excised and digested with trypsin. The resulting peptide mixtures were analyzed using an LTQ Orbitrap Elite Hybrid Mass Spectrometer (Thermo Fisher Scientific, Waltham, MA). The peptide data were analyzed using Mascot MS/MS Ion Search (Matrix Science, Boston, MA) under the following conditions: peptide mass tolerance, 20 parts per million; fragment MS/MS tolerance, 1.2 daltons; allowed up to one missed cleavage; peptide charges, 2^+^, 3^+^, and 4^+^.

### RNA sequencing (RNA-seq) analysis

Sample preparation and RNA-seq analysis were performed as previously described [13]. Briefly, total RNA from CD4^+^ T cells was extracted using TRIzol reagent (catalog no. 15596026; Invitrogen), and 1 µg of total RNA was used for constructing sequencing libraries. RNA libraries for RNA sequencing (RNA-seq) analysis were prepared using the SureSelect XT HS2 mRNA Library Prep Kit (Agilent, Santa Clara, CA). Library samples were analyzed on an Illumina NovaSeq 6000 sequencer (150 bp paired-end reads and dual index configuration). Quality control was performed using Trimmomatic software (v0.36). Reads were aligned to the mouse reference GRCm38 using HISAT2. Feature counts were normalized using the DESeq2 package (v1.28.1). Principal component (factoextra ver.1.0.3; free software) and Gene Ontology enrichment analyses (clusterProfiler ver.3.6; free software) were performed using R. The data generated in this study were deposited in the Gene Expression Omnibus of the National Center for Biotechnology Information [17] and are accessible through the accession number GSE222519 (https://www.ncbi.nlm.nih.gov/geo/query/acc.cgi?acc=GSE222519).

### Statistical analysis

Densitometric analysis of the immunoblotting results was performed using Image-Pro Plus software (Media Cybernetics). All statistical analyses were performed using the Prism 9.0 (GraphPad Software, San Diego, CA). Group differences were analyzed using non-parametric Mann–Whitney U test or two-tailed Student’s t test. Statistical significance was defined at *p* < 0.05.

## Results

### TRAIL-R transduces a negative signal to inhibit proximal TCR singling and T cell activation

**Fig. 1:**
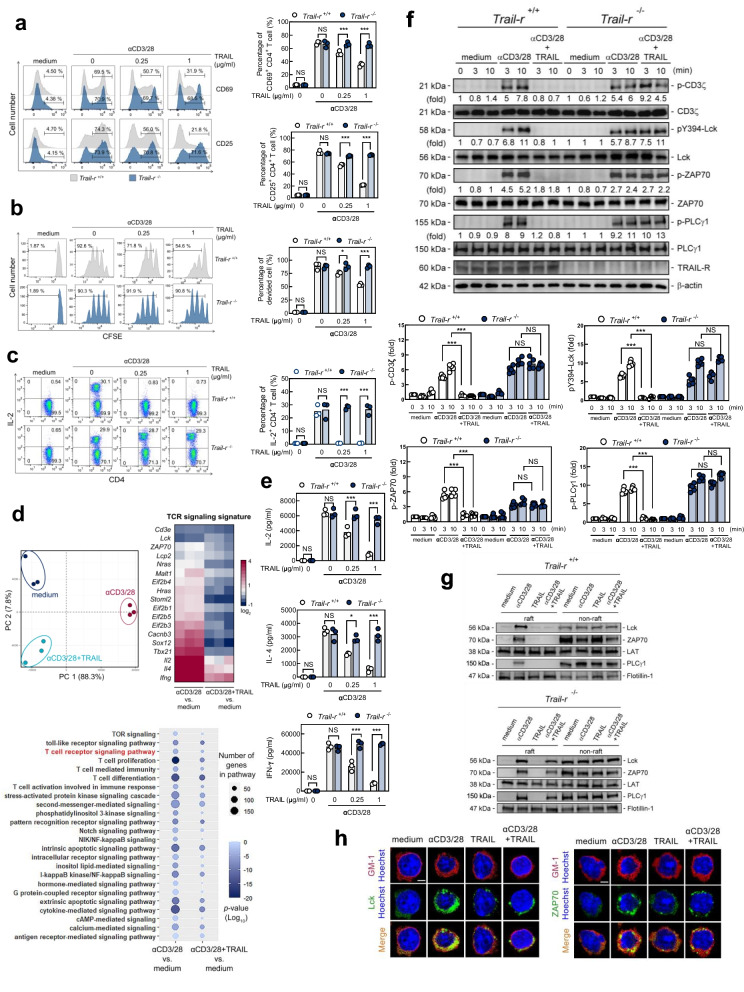
TRAIL-R inhibits proximal TCR signaling and T cell activation. **a** Flow cytometry analyses of T cell activation markers**—**CD69 and CD25 (24 h), (**b**) carboxyfluorescein diacetate succinimidyl diester dilution assays (96 h), and (**c**) intracellular staining of IL-2 (24 h) on *Trail-r*^+/+^ or *Trail-r*^–/–^ murine splenic CD4^+^ T cells stimulated with anti-CD3 (3 µg/ml) and anti-CD28 (2 µg/mL) Abs in the presence/absence of TRAIL (0–1 µg/mL). Data in **a–c** represent analyses from three independent experiments; statistical significance determined by Mann–Whitney U test; NS, no significance; * *p* < 0.05; *** *p* < 0.001. **d** *Trail-r*^+/+^ murine splenic CD4^+^ T cells were stimulated with CD3 (3 µg/mL) and CD28 (2 µg/ml) Abs in the presence/absence of TRAIL (10 µg/mL) for 24 h. Total RNA was extracted and sequenced on the Illumina platform. Principal component analysis represented correlations among groups. Network analysis of biological process Gene Ontology term enrichment among significant genes in the indicated comparisons. Node color is proportional to *p*-adjusted enrichment value; node size proportional to number of genes in each Gene Ontology term. Heatmap showing two differentially expressed genes in TCR signaling pathway. Color bars indicate scores of log_2_-fold change for each comparison. **e** ELISAs of lysates from *Trail-r*^+/+^ or *Trail-r*^–/–^ murine splenic CD4^+^ T cells stimulated with CD3 (3 µg/mL) and CD28 (2 µg/ml) Abs in the presence/absence of TRAIL (10 µg/mL) for 24 h. Data represent analyses from three independent experiments; statistical significance determined by Mann–Whitney U test; NS, no significance; * *p* < 0.05; *** *p* < 0.001. **f** Immunoblotting of phosphorylation of TCR tyrosine kinases in *Trail-r*^+*/*+^ or *Trail-r*^−/−^ murine splenic CD4^+^ T cells at indicated time points. Quantification of phosphorylated protein levels are shown at the bottom of the panel. Data represent analyses from at least three independent experiments; statistical significance determined by two-tailed Student’s t test; NS, no significance; *** *p* < 0.001. **g** *Trail-r*^+*/*+^ or *Trail-r*^−/−^ CD4^+^ T cells were stimulated with anti-CD3 (3 µg/mL) and anti-CD28 (2 µg/mL) Abs in presence/absence of TRAIL (10 µg/mL) for 30 min, then lysed and fractionated as raft and non-raft layers; Lck, ZAP70, LAT, and PLCγ1 were immunoblotted from pooled raft or non-raft fractions. **h** Representative confocal images of *Trail-r*^+*/*+^ murine splenic CD4^+^ T cells following staining with anti-GM-1 (red) and anti-Lck (green), or anti-ZAP70 (green) Abs, as indicated. Colocalization of GM-1 with Lck or ZAP70 is indicated by yellow areas. Scale bar: 4 µm

Recent accumulating evidence indicates that TRAIL suppresses autoimmune inflammation without inducing cell apoptosis and suggests that TRAIL directly inhibits T cell activation to regulate immune responses. In a previous study, we demonstrated that T cell-specific deletion of TRAIL-R completely reversed the anti-inflammatory effects of TRAIL on T cell activation and disease induction in mice with experimental autoimmune encephalomyelitis [13]. This revealed that TRAIL suppresses T cell activation, and that autoimmune inflammation requires TRAIL-R on T cells. To determine whether the TRAIL/TRAIL-R interaction could directly inhibit T cell activation, we stimulated wild-type (*Trail-r*^+*/*+^) or TRAIL-R-knockout (*Trail-r*^−/−^) T cells with anti-CD3 and anti-CD28 antibodies (Abs) in the presence of TRAIL. TRAIL profoundly suppressed T cell activation and significantly decreased T cell activation markers CD69 and CD25 (Fig. 1a), cell proliferation (Fig. 1b), and interleukin-2 (IL-2) production (Fig. 1c) in T cells in *Trail-r* ^+/+^, but not in *Trail-r*^−/−^ mice, indicating that the TRAIL/TRAIL-R interaction is capable of directly inhibiting T cell activation. Next, we utilized whole-transcriptome RNA-seq in TRAIL-treated activated T cells to identify gene expression signatures and possible regulatory pathways associated with TRAIL-R signaling in T cell activation. Principal component and Gene Ontology pathway analyses showed that the transcriptomes of these TRAIL-treated activated T cells were represented by markedly downregulated gene expression signatures in downstream TCR signaling pathways (Fig. 1d, e), suggesting that TRAIL-R regulates the TCR signaling pathway and its downstream signature.

To determine whether TRAIL-R transduces signals to interrupt TCR signaling, we assessed the effect of TRAIL on the phosphorylation of TCR-associated tyrosine kinases in activated T cells. TRAIL engagement profoundly decreased phosphorylation of CD3ζ, tyrosine kinases of proximal TCR signaling (Lck, ZAP70) and its downstream kinases (PLCγ1, ERK, p-38, IKKβ) in T cells activated with anti-CD3/CD28 Abs (Fig. 1f and Supplementary Fig. S1). In contrast, these effects were abolished in the T cells from *Trail-r*^−/−^ mice. This indicated that TRAIL-R transduces a negative signal to inhibit proximal TCR signaling but not downstream signaling. Next, we examined the impact of TRAIL on the recruitment of proximal TCR signaling molecules into lipid rafts, which are a key platform for forming immune synapses and transducing proximal downstream TCR signals [18]. Similarly, TRAIL engagement profoundly impeded the recruitment of TCR proximal tyrosine kinases (Lck and ZAP70) to lipid rafts in T cells activated by anti-CD3/CD28 antibodies, whereas these effects were completely abolished in T cells from *Trail-r*^−/−^ mice (Fig. 1g, 1h). Taken together, these results indicate that TRAIL-R transduces a negative signal to inhibit proximal TCR signaling, leading to the suppression of subsequent T cell activation.

### *TRAIL-R inhibits proximal TCR signaling *via* a pathway distinct from conventional apoptosis signaling mediated by caspase-8*

**Fig. 2:**
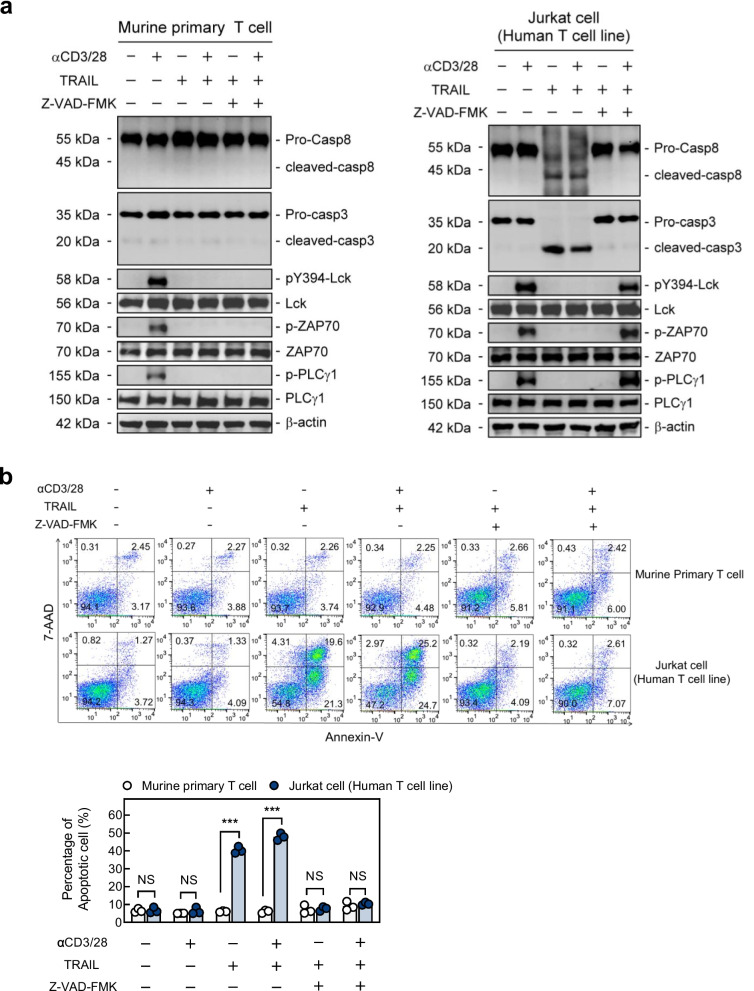
TRAIL-R inhibits proximal TCR signaling without activation of apoptosis signaling. **a** Immunoblotting of caspase-8, caspase-3, and proximal TCR signaling molecules (30 min), and (**b**) Flow cytometry of Annexin V^+^ apoptotic cells (24 h) from murine splenic CD4.^+^ T cells (WT) or Jurkat cells stimulated with anti-CD3 (3 µg/mL) and anti-CD28 (2 µg/mL) Abs with or without TRAIL (10 µg/mL) in the presence or absence of pan-caspase inhibitor, Z-VAD-FMK (10 µg/mL). Data in (**b**) represent analyses from three independent experiments. Statistical significance was determined using the Mann–Whitney U test; NS, no significance; *** *p* < 0.001

TRAIL-R is known to transduce apoptotic signaling via the activation of caspase-8 to induce cell death [19, 20], and whether the signals transduced by TRAIL-R to inhibit TCR signaling also occur via the same pathway remains unclear. Next, we assessed the activation of caspase-8 and its downstream signaling pathways in TRAIL-treated T cells after TRAIL engagement. The results in Fig. 2 revealed TRAIL did not trigger the activation of caspase-8 or caspase-3 in primary T cells. In contrast, both caspase-8 and caspase-3 were fully activated in Jurkat cells (a human T lymphocyte cell line). To further determine whether the caspase-8 pathway is required to inhibit proximal TCR signaling, we examined the activation of these caspases and the phosphorylation of proximal TCR tyrosine kinases in activated T cells during TRAIL engagement in the presence of z-VAD-FMK, a pan-caspase inhibitor. The results revealed that TRAIL profoundly inhibited phosphorylation of proximal TCR tyrosine kinases in the primary T cells and which effect was independent of z-VAD-FMK inhibition (Fig. 2a). Furthermore, as illustrated in Fig. 2b, primary T cells was insensitive to TRAIL-induced cell apoptosis in despite of z-VAD-FMK inhibition, while Jurkat cells were much more sensitive to TRAIL-induced cell apoptosis and z-VAD-FMK could completely block this effect. Taken together, these results indicate that TRAIL-R inhibits proximal TCR signaling via a pathway distinct from that of conventional apoptotic signaling in normal primary T cells.

### SHP-1 associates with TRAIL-R in T cells

**Fig. 3:**
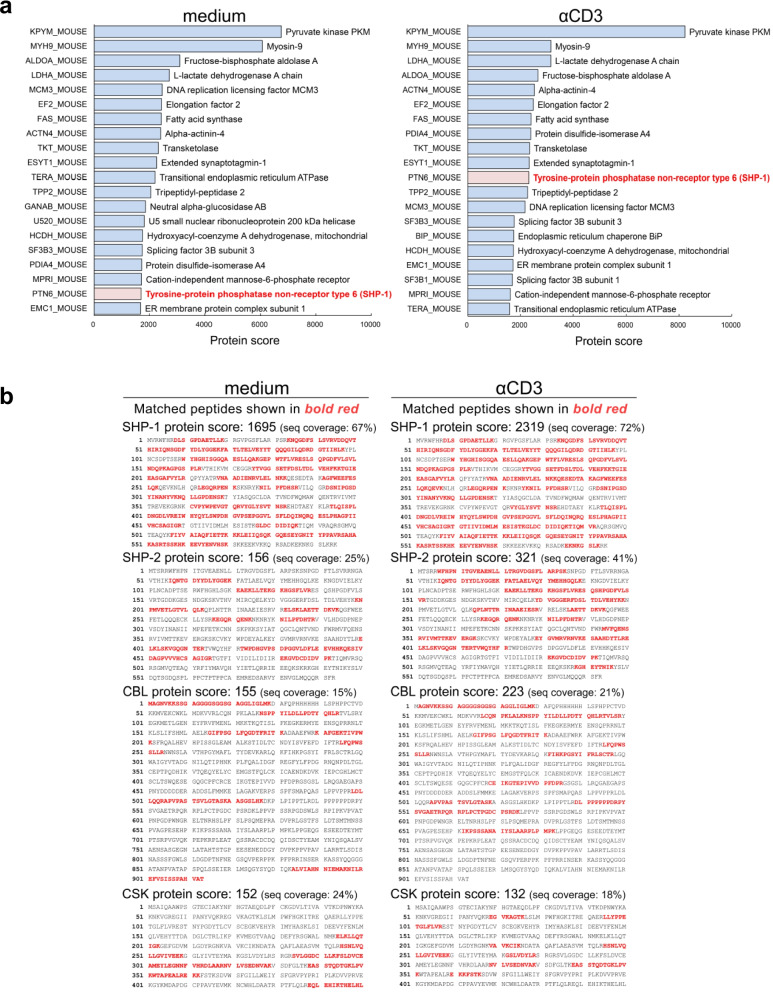
Phosphatase SHP-1 is the major intracellular TRAIL-R binding protein in TRAIL-R IP-MASS proteomics analysis. EL4 cells were transfected with plasmid encoding TRAIL-R, followed by stimulation with anti-CD3 (5 µg/mL) Ab for 30 min. Cell lysates were immunoprecipitated with an anti-TRAIL-R Ab, digested with trypsin, and subjected to LC–MS/MS. Proteins were identified, and scores were mapped using a Mascot MS/MS Ion Search. Protein scores were calculated as the sum of the highest ion scores for each peptide. **a** Ranking of putative TRAIL-R-interacting proteins in the order of protein scores. **b** Protein scores for SHP-1 and other major regulatory proteins involved in proximal TCR signaling

**Fig. 4:**
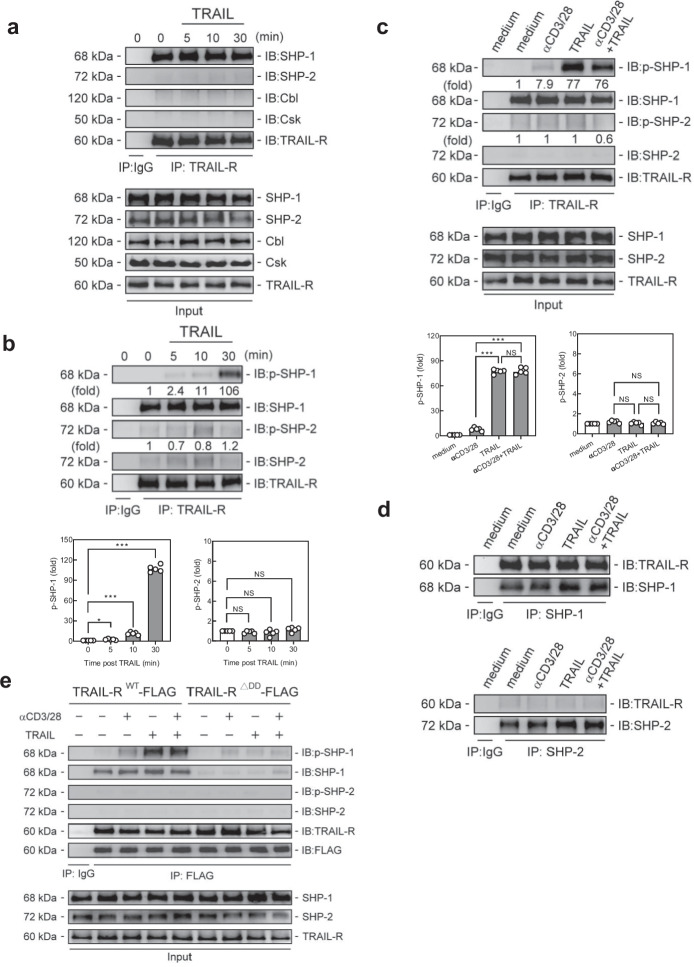
SHP-1 is associated with TRAIL-R in T cells. **a, b** Immunoprecipitation analyses of tyrosine phosphatases SHP-1, SHP-2, Cbl, and Csk with TRAIL-R in murine splenic CD4^+^ T cells in the presence of TRAIL (10 µg/mL) at indicated time point. Quantification of phosphorylated protein levels are shown at the bottom of the panel. Data represent analyses from at least three independent experiments; statistical significance determined by two-tailed Student’s t test; NS, no significance; * *p* < 0.05; *** *p* < 0.001. **c, d** Co-immunoprecipitation of TRAIL-R with SHP-1 or SHP-2 from murine splenic CD4^+^ T cells stimulated with anti-CD3 (3 µg/mL) and anti-CD28 (2 µg/mL) Abs in the presence or absence of TRAIL (10 µg/mL) for 30 min. Quantification of phosphorylated protein levels are shown at the bottom of the panel. Data represent analyses from at least three independent experiments; statistical significance determined by two-tailed Student’s t test; NS, no significance; *** *p* < 0.001. **e** Immunoprecipitation analyses of TRAIL-R with SHP-1 or SHP-2 in TRAIL-R^WT^-FLAG or TRAIL-R^△DD^-FLAG T cells stimulated with anti-CD3 (3 µg/mL) and anti-CD28 (2 µg/mL) Abs in the presence or absence of TRAIL (10 µg/mL) for 30 min

To identify the distinct signaling pathway transduced by TRAIL-R to inhibit proximal TCR signaling, we screened for TRAIL-R binding proteins within T cells for novel TRAIL-R adaptor proteins to transduce signals via immunoprecipitation with TRAIL-R Ab to pull down the TRAIL-R-associated proteins, coupled with mass spectrometry–based proteomics analysis to identify possible candidates. We identified 4015 putative TRAIL-R-interacting proteins in T cells from the anti-TRAIL-R Ab pulldown using mass spectrometry–based proteomics analysis (Supplementary Table S2). Based on the protein score among the TRAIL-R interacting proteins, we identified tyrosine phosphatase SHP-1 as the most likely candidate owing to its high protein score and ability to regulate TCR signaling (Fig. 3a). Furthermore, compared to other principal regulatory proteins involved in proximal TCR signaling, such as SHP-2 (tyrosine phosphatase), Cbl (E3 ubiquitin ligase), and Csk (Src tyrosine kinase). SHP-1 had a much higher protein score with TRAIL-R, suggesting that it could be a novel TRAIL-R-associated protein in T cells (Fig. 3b). Next, we verified the intracellular association between SHP-1 and TRAIL-R in T cells using reciprocal co-immunoprecipitation (Fig. 4). In T cells, SHP-1, but not other TCR signaling-related phosphatases (SHP-2), or negative regulator proteins (Cbl, and Csk), co-immunoprecipitated with anti-TRAIL-R Ab (Fig. 4a). This indicated that SHP-1 is the major intracellular tyrosine phosphatase associated with TRAIL-R in T cells. Additionally, when SHP-1 was associated with TRAIL-R, TRAIL engagement enhanced SHP-1 phosphorylation (Fig. 4b). The association between SHP-1 and TRAIL-R occurs not only in resting T cells but also when T cells are activated with anti-CD3/CD28 antibodies (Fig. 4c, d). To further determine the SHP-1 binding sites at TRAIL-R, we generated the TRAIL-R mutant with truncated death domain (TRAIL-R^△DD^-FLAG), and to analyze the association and phosphorylation of SHP-1 in T cells with full-length (TRAIL-R^WT^-FLAG) or truncated (TRAIL-R^△DD^-FLAG) TRAIL-Rs (Fig. 4E). The results revealed that the association with SHP-1 was completely abolished in TRAIL-R^△DD^-FLAG T cells, indicating SHP-1 binds to TRAIL-R at its death domain in T cells, leading to transducing signals.

### *TRAIL-R-SHP-1 inhibits proximal TCR signaling *via* dephosphorylation of Lck*

**Fig. 5:**
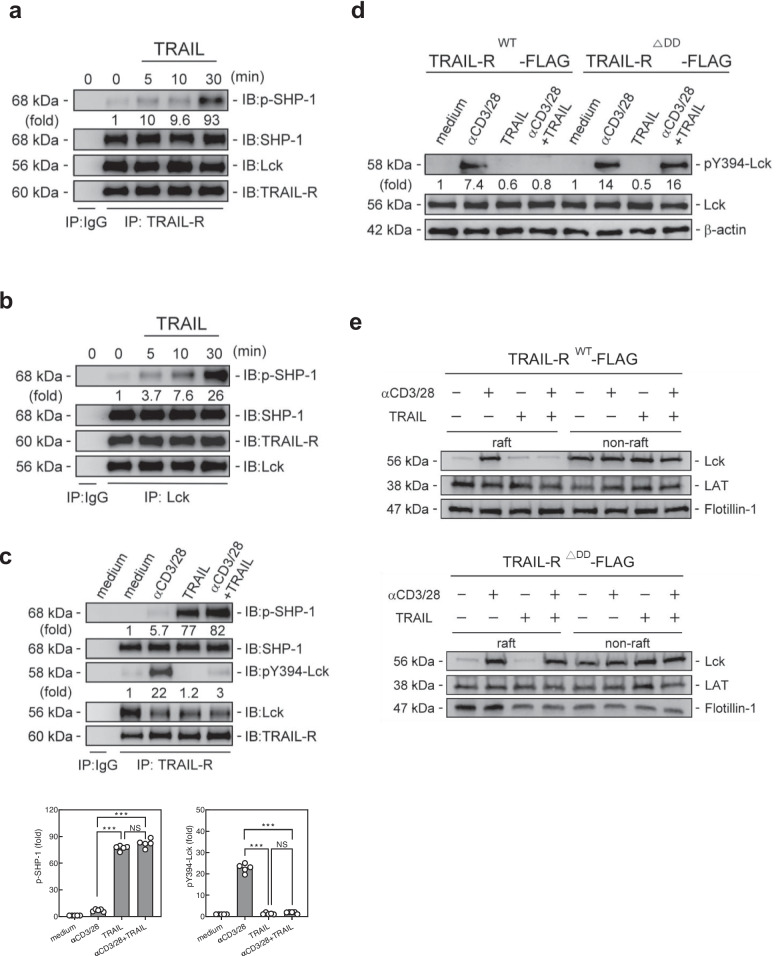
TRAIL-R/SHP-1 inhibits proximal TCR signaling and T cell activation via dephosphorylation of Lck. **a, b** Immunoprecipitation with anti-TRAIL-R **(a)** or anti-Lck **(b)** Ab and then immunoblotting with p-SHP-1 and SHP-1 in murine splenic CD4^+^ T cells treated with TRAIL (10 µg/mL) at indicated time points. Quantification of phosphorylated protein levels are shown at the bottom of the panel. **c** Immunoprecipitation with anti-TRAIL-R Ab and then immunoblotting with p-Lck (Y394) and Lck in murine splenic CD4^+^ T cells stimulated with anti-CD3 (3 µg/mL) and anti-CD28 (2 µg/mL) Abs in the presence or absence of TRAIL (10 µg/mL) for 30 min. Quantification of phosphorylated protein levels are shown at the bottom of the panel. Data represent analyses from at least three independent experiments; statistical significance determined by two-tailed Student’s t test; NS, no significance; *** *p* < 0.001. **d, e** Immunoblotting of Lck in lysates (**d**) and pooled raft or non-raft fractions (**e**) of TRAIL-R^WT^-FLAG or TRAIL-R^△DD^-FLAG T cells stimulated with anti-CD3 (3 µg/mL) and anti-CD28 (2 µg/mL) Abs in the presence or absence of TRAIL (10 µg/mL) for 30 min. Quantification of phosphorylated protein levels are shown at the bottom of the panel

To further determine the possible molecular targets of TRAIL-R/SHP-1 in the regulation of proximal TCR signaling, we assessed the impact of TRAIL-R/SHP-1 on the phosphorylation of Lck at Y394, where SHP-1 specifically dephosphorylates and inactivates Lck [21] to halt proximal TCR signaling [22]. As shown in Fig. 5, Lck co-immunoprecipitated with the TRAIL-R/SHP-1 complex in T cells, indicating that they could form a TRAIL-R/SHP-1/Lck complex in T cells (Fig. 5a, b), accompanied with the enhanced SHP-1 phosphorylation under TRAIL engagement. In addition, when T cells were activated with anti-CD3/CD28 antibodies, Lck (Y394) was induced in the TRAIL-R/SHP-1/Lck complex. TRAIL engagement significantly reduced Lck (Y394) phosphorylation in the activated T cells along with the increased phosphorylated SHP-1 in the TRAIL-R/SHP-1/Lck complex (Fig. 5c). These results indicate TRAIL-R/SHP-1 induces dephosphorylation of Lck at Y394, leading to interruption of proximal TCR signaling. Next, we examined whether this effect was dependent on the binding of SHP-1 to the death domain of TRAIL-R in T cells. The results revealed that TRAIL engagement inhibited the phosphorylation of Lck (Y394) only in TRAIL-R^WT^-FLAG T cells but not in TRAIL-R^△DD^-FLAG T cells when activated with anti-CD3/CD28 Abs (Fig. 5d). Moreover, TRAIL engagement profoundly suppresses the recruitment of Lck to lipid rafts during T cell activation. In contrast, all these inhibitory effects of TRAIL were abolished in TRAIL-R^△DD^-FLAG T cells (Fig. 5e), indicating the binding of SHP-1 to TRAIL-R at its death domain in T cells induces Lck at Y394 dephosphorylation, which in turn inhibits proximal TCR signaling.

**Fig. 6:**
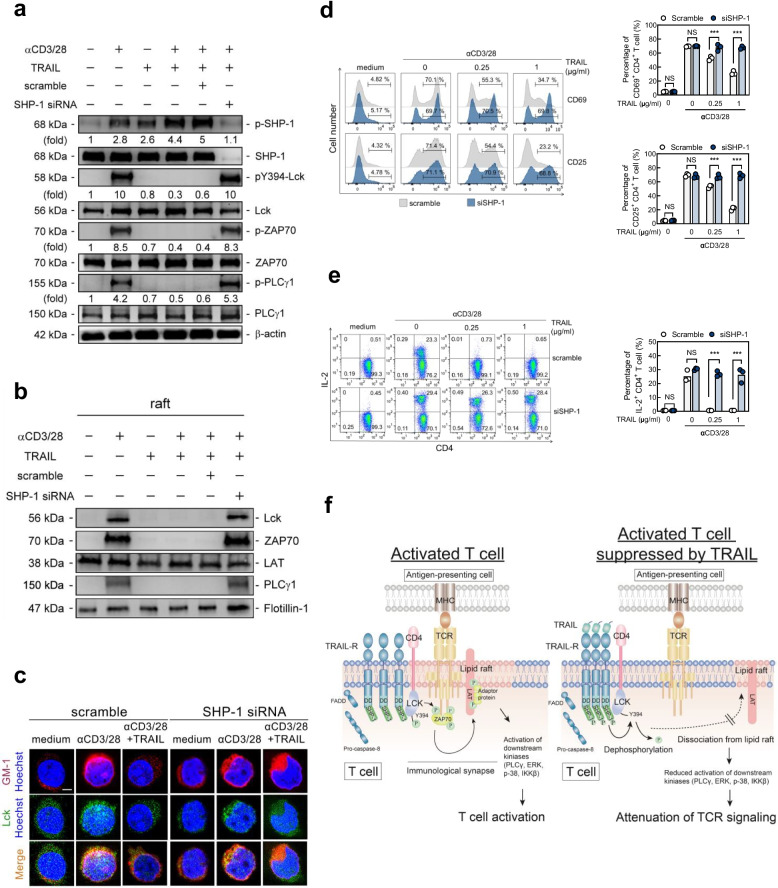
SHP-1 knockdown abolished TRAIL-R-mediated inhibition on Lck phosphorylation, lipid raft recruitment and T cell activation. **a** Immunoblotting of phosphorylation of Lck and other proximal TCR signaling molecules (30 min), (**b**) immunoblotting of Lck and other proximal TCR signaling molecules in pooled raft fractions (30 min), (**c**) representative confocal images of co-localization of GM-1 with Lck (30 min), (**d**) flow cytometry of T cell activation markers CD69 and CD25 (24 h) (Scale bar, 4 µm), and (**e**) intracellular staining of IL-2 from murine splenic CD4.^+^ T cells transfected with scramble or SHP-1 siRNA, followed by stimulation with anti-CD3 (3 µg/mL) and anti-CD28 (2 µg/mL) Abs in the presence/absence of TRAIL (0–1 µg/mL) for 24 h. Quantification of phosphorylated protein levels are shown at the bottom of the panel. Data **d–e** represent analyses from three independent experiments and statistics determined using Mann–Whitney U test. NS, not significant; *** *p* < 0.001. **f** Model of TRAIL-R/SHP-1 repressing TCR signaling and T cell activation through dephosphorylation of Lck at Y394. Upon TCR ligation, Lck binds to, and phosphorylates TCR and Zap70 to form an immunological synapse in the lipid raft, which transduces proximal TCR signaling downstream, resulting in T cell activation (Left). TRAIL engagement reduces Lck (Y394) phosphorylation along with the increased phosphorylated SHP-1 in the TRAIL-R/SHP-1/Lck complex, leading to the dissociation of the co-receptor-Lck complex from the lipid raft, which in turn limits downstream TCR signaling to restrain T cell activation (Right)

In order to confirming the role of TRAIL-R/SHP1/Lck axis in proximal TCR signaling and T cell activation, we further used siRNA to knockdown SHP-1 expression to access its impact on phosphorylation of Lck and TCR signaling. As the results illustrated in Fig. 6, SHP-1 knockdown nearly completely abolished the inhibitory effects of TRAIL on phosphorylation of the TCR proximal signaling kinases Lck, ZAP-70, and PLCγ1 (Fig. 6a) as well as the recruitment of these signaling molecules into lipid raft (Fig. 6b, c) and the subsequent T cell activation (Fig. 6d, e). Taken together, these results indicate that TRAIL-R/SHP-1 transduces co-inhibitory signaling to inhibit phosphorylation of proximal TCR tyrosine kinases via inactivation of Lck, which leads to the dissociation of the co-receptor-Lck complex in the lipid raft and in turn limits the subsequent signaling downstream of the TCR to inhibit T cell activation and avoid autoimmunity (Fig. 6f).

## Discussion

In this study, we showed that TRAIL-R binds to the phosphatase SHP-1 and transduces a co-inhibitory signaling pathway distinct from the conventional apoptosis signaling pathway to inhibit proximal TCR signaling and subsequent T cell activation via inactivation of Lck. It defines TRAIL-R as a new class of immune checkpoint receptor involved in the regulation of autoimmunity.

TRAIL-R transduces apoptotic signaling after binding to its ligand TRAIL [3, 4] and activates caspase-8 and the downstream caspase cascade [5]. Accumulating evidence has demonstrated the emerging role of TRAIL in regulating immune responses and T cell homeostasis in autoimmune diseases [6–10]. Previous studies have attributed TRAIL’s anti-inflammatory effects of TRAIL to its role in inducing immune cell death; however, recent studies have demonstrated that TRAIL inhibits T cell activation and suppresses autoimmune inflammation without inducing cell apoptosis in mouse animal models [11–13]. Here, we demonstrated that TRAIL engagement directly inhibits TCR signaling and subsequent T cell activation without inducing apoptosis. However, it remains unclear how TRAIL-R transduces signaling, a pathway distinct from apoptotic signaling, to directly inhibit TCR signaling.

TRAIL induces cell apoptosis in many tumor cell lines, including Jurkat cells, but not in most primary cells, indicating the apoptosis signaling is tightly regulated in primary T cells to prevent cell death by extrinsic stimulation. Interaction of TRAIL with the TRAIL-R induces recruitment of the FADD, which recruits pro-caspase-8 and to form the death-inducing signaling complex (DISC), in turn activates the caspase cascade and eventually leads to cell death. It is known that the DISC assembly is tightly regulated by FLIP, which is the most critical negative regulators of Fas and TRAIL apoptosis signaling. c-FLIP competes with caspase-8 for binding to the adaptor protein FADD following death receptors' (DRs) activation via the ligands of the TNF-R family. Therefore, TRAIL is able to induce strong cell apoptosis in Jurkat cells, but failed to induce apoptosis in CD4^+^ T cells. When TRAIL engagement is not able to trigger effective apoptosis signaling in CD4^+^ T cells, it could activate the alternative signaling pathway. Here we identified the TRAIL-R/SHP-1/Lck axis, which transduces co-inhibitory signal to inhibit proximal TCR signaling and subsequent T cell activation**—**a novel pathway distinct from the conventional apoptosis signaling pathway. It appears that the TRAIL-R/SHP-1/Lck complex exits in both murine primary CD4^+^ (Fig. 5) and CD8^+^ T cells (Supplementary Fig. S2a) as well as the human T cell line, Jurkat cells (Supplementary Fig. S2b); only primary T cells are resistant to TRAIL-induced apoptosis. Therefore, in spite of existence of TRAIL-R/SHP-1/Lck complexes, TRAIL engagement triggers efficient cell apoptosis, instead of activating TRAIL-R/SHP-1/Lck axis in Jurkat cells.

In fact, in consistent with the findings in murine primary CD4^+^ T cells, TRAIL profoundly suppressed T cell proliferation and interleukin-2 (IL-2) production in human CD4^+^ T cells from patient with autoimmune diseases (Supplementary Fig. S3). In addition, TRAIL engagement profoundly decreased phosphorylation of Lck in human CD4^+^ T cells activated the anti-CD3/CD28 Abs (Supplementary Fig. S4). All these results implicate the immuno-regulatory role of TRAIL-R on regulation of T cell activation in human autoimmune diseases.

Our results identified the tyrosine phosphatase SHP-1 as the major TRAIL-R-associated protein in T cells, and the TRAIL-R/SHP-1 complex was capable of transducing signals when engaged with TRAIL. Moreover, TRAIL engagement induces globally decreased phosphorylation of tyrosine kinases involved in TCR proximal signaling and immune synapse formation during T cell activation. These findings suggest that TRAIL-R transduces a negative signal to inhibit TCR signaling via association with SHP-1. In T cells, it has been demonstrated SHP-1 inhibits several TCR-induced pathways in T cells, including cytokine production and proliferation [23, 24]. In fact, motheaten mice with SHP-1 gene mutations exhibit enhanced TCR signaling with autoimmunity and profound chronic inflammation [25]. Here, we demonstrate that SHP-1 is an important phosphatase and that the TRAIL-R/SHP-1 complex transduces signals to inactivate Lck, leading to the attenuation of TCR signaling and subsequent T cell activation. A recent investigation on the effects of immune checkpoint receptors on T cells revealed that these inhibitory receptors, such as PD-1, act by negatively modulating the network of pre-existing TCR signaling, particularly proximal TCR signaling [26]. In T cells, PD-1 signaling relies mainly on tyrosine phosphatase SHP-2 [27, 28]. Although the proteomics results in this study identified that SHP-1 is associated with TRAIL-R in T cells, it does not completely preclude a role for SHP-2. In addition, our results revealed that SHP-1 binds to TRAIL-R at the death domain, which is the same binding site where the apoptosis signaling complex is assembled. This suggests that SHP-1 competes with caspase-8 for binding to TRAIL-R and preventing apoptotic signaling, which is similar to the interactions observed with several inhibitory proteins [29]. Our results suggest that TRAIL-Rs may act as a new class of immune checkpoint receptors in the regulation of autoimmunity. Similar to the results of T cells activated with anti-CD3/CD28, TRAIL engagement is still able to suppress T cell activation when CD4^+^ T cells activated by anti-PD-1 (Supplementary Fig. S5), indicating TRAIL/TRAIL-R interaction is capable of inhibiting anti-PD-1-mediated T cell activation.

TRAIL-R is distinct from the other conventional inhibitory receptors. Conventional inhibitory receptors and checkpoint receptors contain single conserved I/VxYxxL motifs, termed immunoreceptor tyrosine-based inhibitory motifs (ITIMs), that are responsible for the recruitment and activation of inhibitory phosphatases [30, 31]. However, TRAIL-R does not contain ITIMs [32]. This distinct association between phosphatases and death receptors [32, 33] suggests a new class of gatekeeper receptors that regulate TCR signaling. Although in this study, our results implicate TRAIL treatment has effects on the TRAIL-R/SHP-1/Lck complex and associates with the enhanced SHP-1 phosphorylation, it is still not clear the actual role of SHP-1 phosphorylation on Lck activity, and it needs further in-depth exploration to clarify the spatial and kinetic relationships of these microcluster components to address the interaction and crosstalk among TCR complex, Lck, TRAIL-R, and SHP-1.

Lck is an Src-related protein tyrosine kinase that initiates the earliest TCR signaling event by phosphorylating CD3 when an immune synapse forms, which results in the phosphorylation of ZAP70 and in turn recruits proximal TCR signaling molecules into lipid rafts to transduce proximal signals downstream [34]. Lck activation is tightly controlled by the phosphorylation states of Y394 and Y505, which represent activating and inhibitory tyrosine residues, respectively. SHP-1 mediates a negative feedback pathway that dephosphorylates and activates Y394 [21]. In support of the critical role of Lck in regulating TCR signaling, our results indicate that TRAIL-R/SHP-1 transduces signaling to inhibit proximal TCR signaling via the dephosphorylation of Lck. However, our study has some limitations. First, it is still not clear whether TRAIL-R and its coreceptors interact intrinsically owing to the electrostatically driven protein–protein interactions. Second, although our proposed TRAIL-R/SHP-1/Lck axis is supported by multiple biochemical and cellular functional approaches, the spatial interactions of these molecules remain unclear. Third, the current experimental designs used anti-CD3/28 Abs instead the real MHC-mounted antigen to trigger T cell activation. Future analyses are required to clarify the spatial and kinetic relationships of these microcluster components using super-resolution imaging [35] to address the interaction and crosstalk among TCR complex, Lck, TRAIL-R, and SHP-1.

## Conclusions

TRAIL-R/SHP-1 transduces a distinct immune gatekeeper signaling pathway to regulate TCR signaling via direct inactivation of Lck, leading to reduced T cell activation. Thus, our results define TRAIL-R as a new class of immune checkpoint receptors for restraining T cell activation, and TRAIL-R/SHP-1 axis can serve as a potential therapeutic target for immune-mediated diseases.

### Supplementary Information

**Supplementary Material 1.**

**Supplementary Material 2.**

**Supplementary Material 3.**

## Data Availability

All data supporting the findings of this study are available within the paper and its Supplementary Information.

## Abbreviations

LC–MS/MS: Liquid chromatography–tandem mass spectrometry
RNA-seq: RNA sequencing
TCR: T cell receptor
TNF: Tumor necrosis factor
TRAIL-R: TNF-related apoptosis-inducing ligand receptor
