# Supplementary material for: Association of TRAIL receptor with phosphatase SHP-1 enables repressing T cell receptor signaling and T cell activation through inactivating Lck
Source: J Biomed Sci. 2024 Mar 27;31:33. doi: 10.1186/s12929-024-01023-8 (PMC10967194; doi:10.1186/s12929-024-01023-8)
Supplement: Supplementary file 1 — Supplementary Material 1. [file 12929_2024_1023_MOESM1_ESM.pdf]

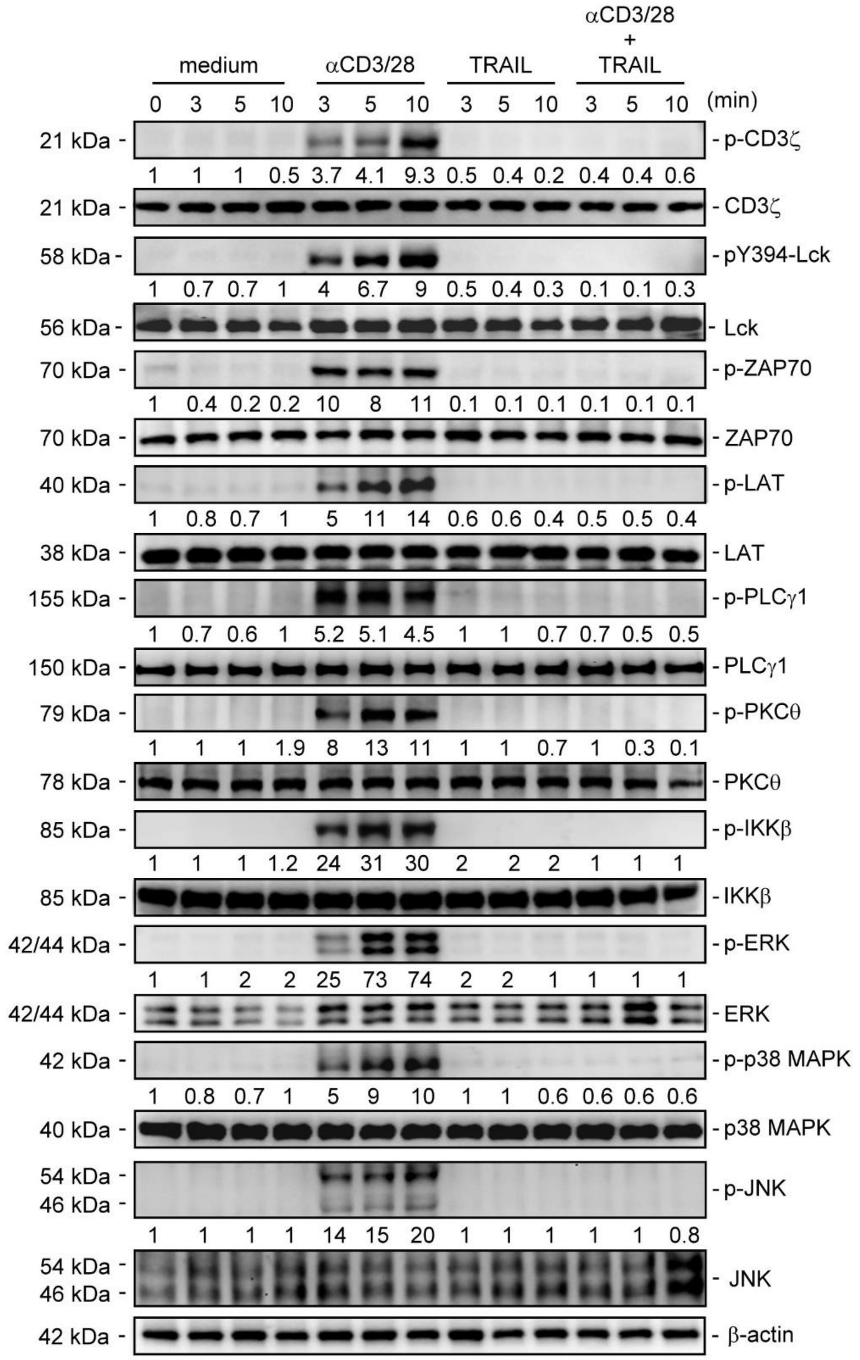

Fig. S1 TRAIL inhibits phosphorylation of tyrosine kinases of proximal TCR signaling and its downstream kinases.

Immunoblotting of TCR signaling molecules in murine splenic CD4^{+} T cells stimulated with anti-CD3 (3 μg/ml) and anti-CD28 (2 μg/ml) antibodies in the presence or absence of TRAIL (10 μg/ml) at indicated time point. Quantification of phosphorylated protein levels are shown at the bottom of the panel.

a

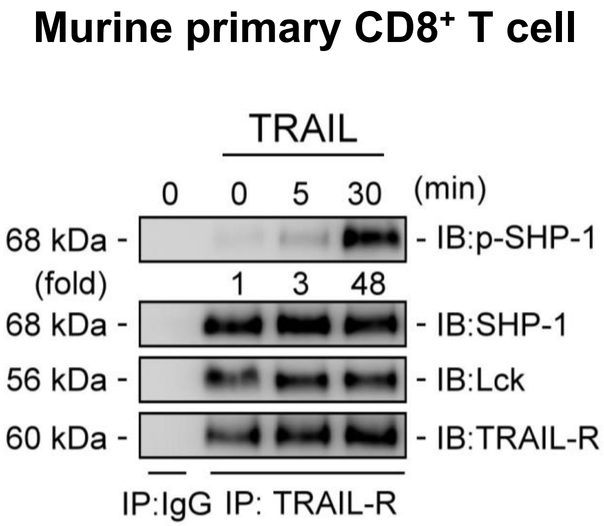

b

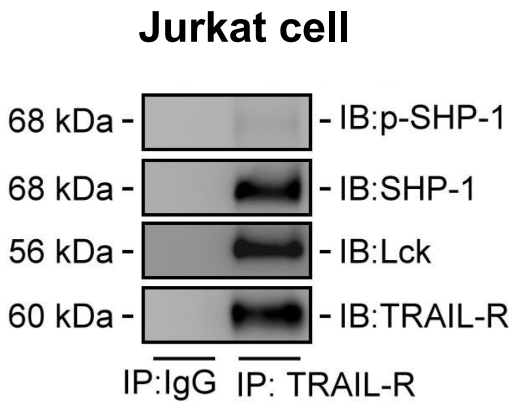

Fig. S2 TRAIL-R/SHP-1/Lck complexes exist in murine CD8$^+$ T cells and Jurkat cells. (**a**) Murine splenic CD8 T cells were isolated by MojoSort™ Mouse CD8 T Cell Isolation Kit (BioLegend, catalog no. 480008). Immunoprecipitation with anti-TRAIL-R and then immunoblotting with p-SHP-1 and SHP-1 in murine splenic CD8$^+$ T cells treated with TRAIL (10 μg/mL) at indicated time points. (**b**) Immunoprecipitation with anti-TRAIL-R Ab and then immunoblotting with p-SHP-1 and SHP-1 in Jurkat cells.

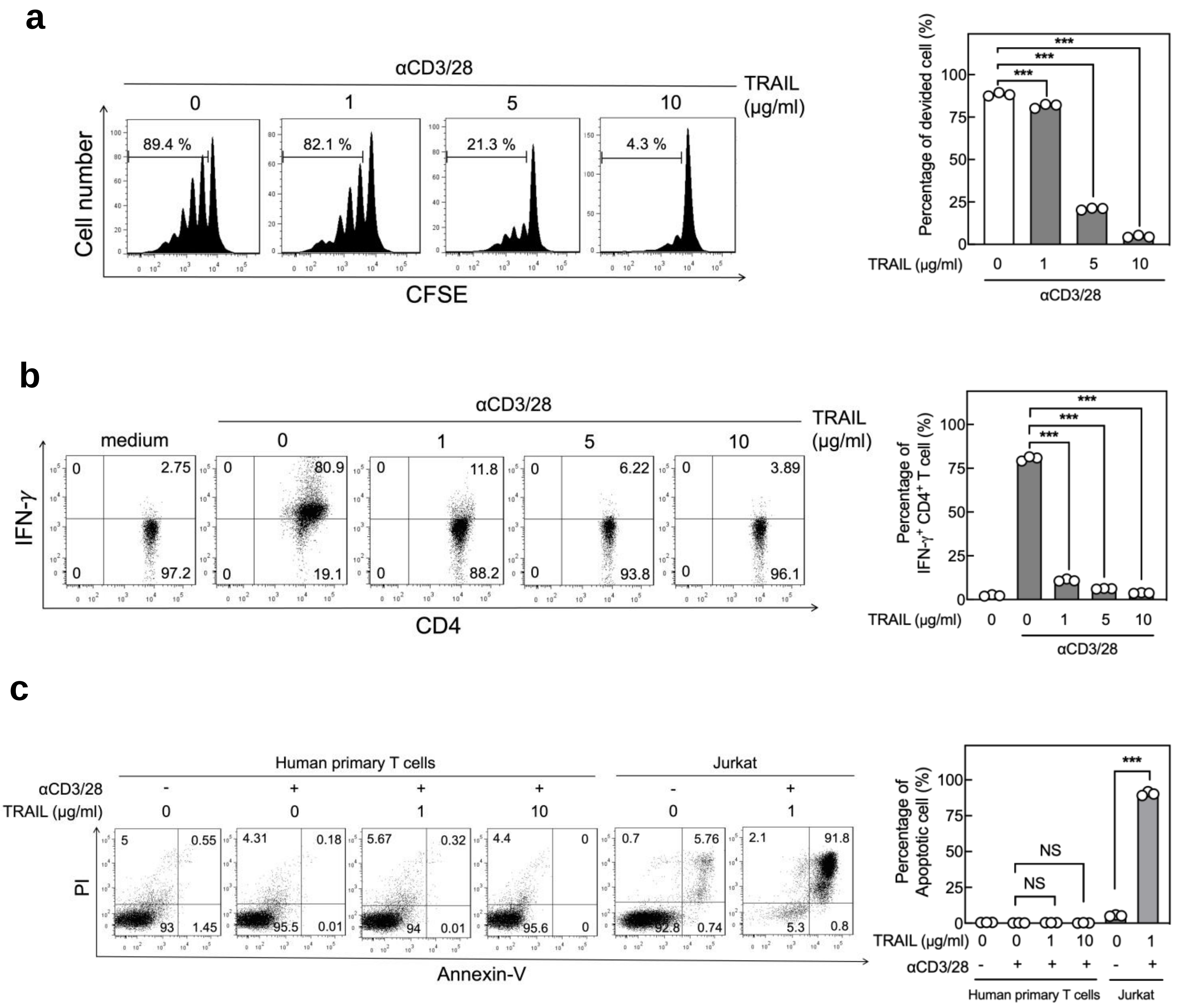

Fig. S3 TRAIL inhibits proliferation and cytokine production without inducing cell apoptosis in T cells from patients with systemic lupus erythematosus (SLE).
Human CD4 T cells were isolated from peripheral blood mononuclear cell (PBMC) from SLE patients by RosetteSep Human CD4$^+$ T cell Enrichment Cocktail (STEMCELL Technologies, catalog no. 17107661). (**a**) Carboxyfluorescein diacetate succinimidyl diester dilution assays (96 h), (**b**) intracellular staining of IFN-γ (BioLegend, catalog no. 506510) (24 h), and (**c**) flow cytometry of Annexin V$^+$ apoptotic cells (24 h) from the human CD4$^+$ T cells or Jurkat cells were stimulated with anti-CD3 (3 μg/ml) (BioLegend, catalog no. 317347) and anti-CD28 (2 μg/ml) (BioLegend, catalog no. 302943) antibodies in the presence of TRAIL (PeproTech, catalog no. #310-04-250UG) at the indicated concentration. Data in **a–c** represent analyses from three independent experiments; statistical significance determined by Mann–Whitney U test; NS, no significance; * ***$p < 0.001$. All studies that included samples from human subjects were conducted in accordance with institutional guidelines (Far Eastern Memorial Hospital, Permission No.: 107002-E).

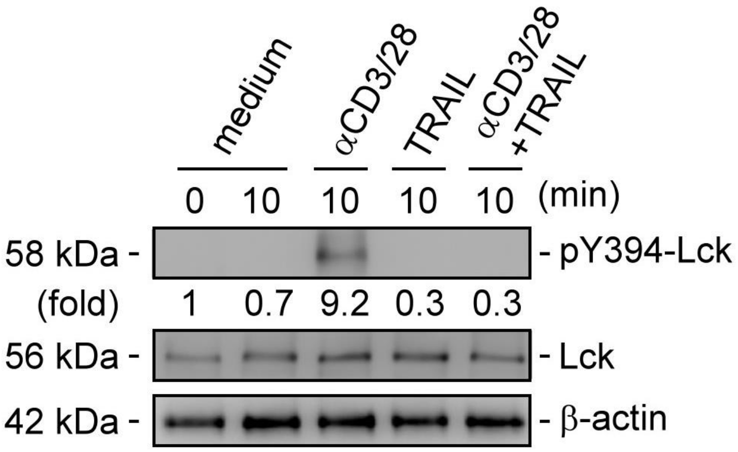

Fig. S4 TRAIL inhibited phosphorylation of Lck in human T cells from patients with systemic lupus erythematosus (SLE).

Human T cells were isolated from peripheral blood mononuclear cell (PBMC) from SLE patients by RosetteSep Human $CD4^+$ T cell Enrichment Cocktail (STEMCELL Technologies, catalog no. 17107661). Immunoblotting of pY394-Lck (BioLegend, catalog no. 933102, Clone: A18002D), Lck (Cell signaling, catalog no. 2752) and β-actin (Merck Millipore, catalog no. MAB1501) in human T cells were stimulated with anti-CD3 (3 µg/ml) (BioLegend, catalog no. 317347) and anti-CD28 (2 µg/ml) (BioLegend, catalog no. 302943) antibodies in the presence of TRAIL (10 µg/ml) (PeproTech, catalog no. #310-04-250UG) at indicated time point. Quantification of pY394-Lck level is shown at the bottom of the panel.

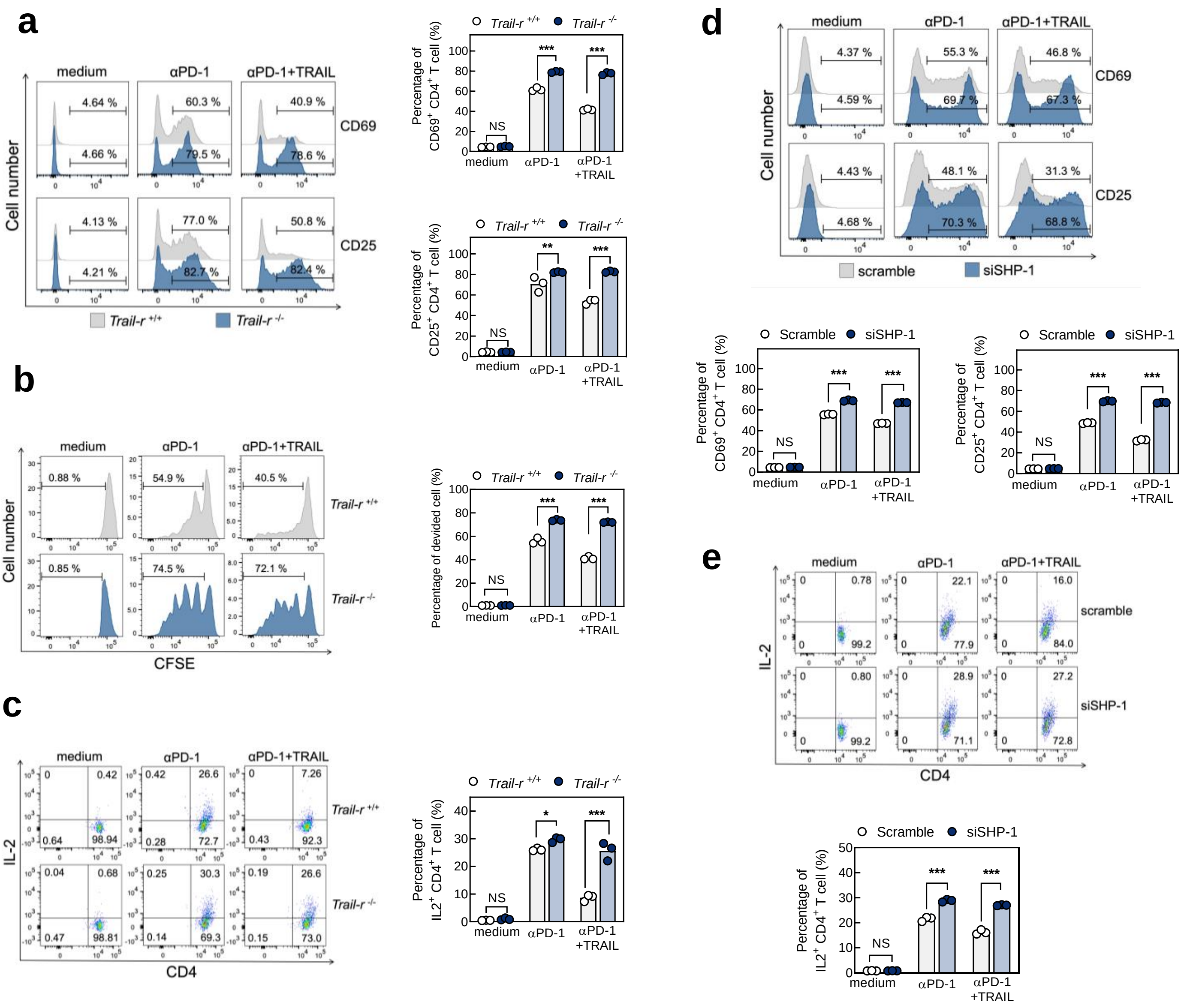

Fig. S5 TRAIL/TRAIL-R interaction inhibited PD-1 blockade-mediated T cell activation while SHP-1 knockdown abolished the inhibition.

(**a–c**) Murine splenic CD4$^+$ T cells were pre-treated with anti-CD3 (3 µg/ml) and anti-CD28 (2 µg/ml) Abs for 24 h, followed by stimulation with anti-PD-1 (10 µg/ml) Ab (Bio X Cell, catalog no. BE0033-2; New Hampshire, Lebanon) in the presence/absence of TRAIL (10 µg/mL) for another 24 h. Flow cytometry analyses of T cell activation markers of CD69 and CD25 (24 h) (**a**), carboxyfluorescein diacetate succinimidyl diester dilution assays (96 h) (**b**) , and Intracellular staining of IL-2 (24 h) (**c**) were assayed. (**d–e**) Murine splenic CD4$^+$ T cells were transfected with scramble or SHP-1 siRNA, pre-treated with anti-CD3 (3 µg/ml) and anti-CD28 (2 µg/ml) Abs for 24 h, followed by stimulation with anti-PD-1 (10 µg/ml) Ab in the presence/absence of TRAIL (10 µg/mL) for another 24 h. Flow cytometry of T cell activation markers of CD69 and CD25 (24 h) (**d**), and intracellular staining of IL-2 (**e**) were assayed. Data represent analyses from three independent experiments and statistics determined using Mann–Whitney U test. NS, not significant; *$p < 0.05$; ***$p < 0.001$.
