# Supplementary material for: Association of TRAIL receptor with phosphatase SHP-1 enables repressing T cell receptor signaling and T cell activation through inactivating Lck
Source: J Biomed Sci. 2024 Mar 27;31:33. doi: 10.1186/s12929-024-01023-8 (PMC10967194; doi:10.1186/s12929-024-01023-8)
Supplement: Supplementary file 2 — Supplementary Material 2. [file 12929_2024_1023_MOESM2_ESM.docx]

**Supplementary Table S1 List of antibodies used for immunoblotting and immunoprecipitation**

| **Antibody** | **Clone** | **Host** | **Catalog No** | **Company** | **Application** |
| --- | --- | --- | --- | --- | --- |
| LCK | polyclonal | rabbit | 2752S | Cell signaling | WB, IP |
| ZAP70 | D1C10E | rabbit | 3165S | Cell signaling | WB |
| p-ZAP70 (Y319/Y352) | 65E4 | rabbit | 2717S | Cell signaling | WB |
| PLCγ1 | D9H10 | rabbit | 5690 | Cell signaling | WB |
| p-PLCγ1 (Y783) | polyclonal | rabbit | 2821S | Cell signaling | WB |
| p-LAT (Y255) | polyclonal | rabbit | 45170 | Cell signaling | WB |
| PKCθ | E117Y | rabbit | 13643 | Cell signaling | WB |
| p-PKCθ (T538) | polyclonal | rabbit | 9377 | Cell signaling | WB |
| p-IKKβ (S176/180) | monoclonal | rabbit | 2697S | Cell signaling | WB |
| JNK | polyclonal | rabbit | 9252 | Cell signaling | WB |
| p-JNK (T183/Y185) | polyclonal | rabbit | 9251 | Cell signaling | WB |
| p38 MAPK | polyclonal | rabbit | 9212 | Cell signaling | WB |
| Caspase 8 | 11G10 | mouse | 9748S | Cell signaling | WB |
| p-SHP-1 (Y564) | D11G5 | rabbit | 8849S | Cell signaling | WB |
| SHP-2 | D50F2 | rabbit | 3397S | Cell signaling | WB, IP |
| p-SHP-2 (Y580) | polyclonal | rabbit | 3703S | Cell signaling | WB |
| CBL | polyclonal | rabbit | 2747S | Cell signaling | WB |
| CSK | C74C1 | rabbit | 4980 | Cell signaling | WB |
| FLAG (DYKDDDDK-Tag) | polyclonal | rabbit | 2368 | Cell signaling | WB, IP |
| p-LCK (Y394) | A18002D | mouse | 933102 | BioLegend | WB |
| ERK1/2 | W15133B | rat | 686901 | BioLegend | WB |
| p-ERK1/2 (T202/Y204) | 4B11B69 | mouse | 675506 | BioLegend | WB |
| p-CD3ζ (Y142) | polyclonal | rabbit | abx012434 | Abbexa | WB |
| LAT | polyclonal | rabbit | PA5-82419 | Invitrogen | WB |
| TRAIL-R (DR5) | polyclonal | rabbit | 3062 | BioVision | WB, IP |
| CD3ζ | G3 | mouse | ab11281 | Abcam | WB |
| IKKβ | EPR6043 | rabbit | ab124957 | Abcam | WB |
| Caspase 3 | EPR18297 | rabbit | ab184787 | Abcam | WB |
| SHP-1 | polyclonal | rabbit | ab227503 | Abcam | WB, IP |
| p-p38 MAPK (T180/Y182) | polyclonal | rabbit | 28796-1 | Proteintech | WB |
| Flotillin-1 | 18/Flotillin-1 | mouse | 610821 | BD Biosciences | WB |
| β-actin | C4 | mouse | MAB1501 | Merck Millipore | WB |

Abbreviation: WB, immunoblotting; IP, immunoprecipitation.
